# Behavioral evidence for the role of cortical θ oscillations in determining auditory channel capacity for speech

**DOI:** 10.3389/fpsyg.2014.00652

**Published:** 2014-07-04

**Authors:** Oded Ghitza

**Affiliations:** Department of Biomedical Engineering, Hearing Research Center, Boston UniversityBoston, MA, USA

**Keywords:** information transfer rate, auditory channel capacity, fast speech, phonetic variability, intelligibility, brain rhythms, theta oscillations

## Abstract

Studies on the intelligibility of time-compressed speech have shown flawless performance for moderate compression factors, a sharp deterioration for compression factors above three, and an improved performance as a result of “repackaging”—a process of dividing the time-compressed waveform into fragments, called packets, and delivering the packets in a prescribed rate. This intricate pattern of performance reflects the reliability of the auditory system in processing speech streams with different information transfer rates; the knee-point of performance defines the auditory channel capacity. This study is concerned with the cortical computation principle that determines channel capacity. Oscillation-based models of speech perception hypothesize that the speech decoding process is guided by a cascade of oscillations with theta as “master,” capable of tracking the input rhythm, with the theta cycles aligned with the intervocalic speech fragments termed θ-syllables; intelligibility remains high as long as theta is in sync with the input, and it sharply deteriorates once theta is out of sync. In the study described here the hypothesized role of theta was examined by measuring the auditory channel capacity of time-compressed speech undergone repackaging. For all speech speeds tested (with compression factors of up to eight), packaging rate at capacity equals 9 packets/s—aligned with the upper limit of cortical theta, θ_max_ (about 9 Hz)—and the packet duration equals the duration of one uncompressed θ-syllable divided by the compression factor. The alignment of both the packaging rate and the packet duration with properties of cortical theta suggests that the auditory channel capacity is determined by theta. Irrespective of speech speed, the maximum information transfer rate through the auditory channel is the information in one uncompressed θ-syllable long speech fragment per one θ_max_ cycle. Equivalently, the auditory channel capacity is 9 θ-syllables/s.

## 1. Introduction

How human brain circuitry enables our communication capabilities constitutes a compelling scientific challenge. We possess only a rudimentary understanding of neuronal computation, and there are only few hypotheses that link brain mechanisms with elementary cognitive computations that underlie processing sensory input. In the broader context, the study reported here aims at unveiling cortical computational principles that govern *recognition*, using the speech communication mode as a vehicle.

In comprehending spoken language, the listener faces the task of decoding a linguistic message embedded in the acoustic waveform. Since words pronounced by the same speaker—and even more so words pronounced by different speakers—markedly differ in their acoustic realization, the listener is faced with the task of mapping a variant stimulus onto an invariant response. The ease by which we can comprehend speech irrespective of inter-speaker variability—in gender, age, accent, speed, duration—is therefore remarkable. The cortical computational principles that enable such capability are yet to be understood.

A particular phonetic variability of interest is speech speed. Studies on the effects of time compression of speech on intelligibility (e.g., Garvey, 1953; Foulke and Sticht, 1969; Dupoux and Green, 1997; Reed and Durlach, 1998; Versfeld and Dreschler, 2002; Peelle and Wingfield, 2005), have shown flawless performance for moderate compression ratios, but a sharp deterioration in intelligibility for compression ratios above about three (with word error rates greater than 50%). What is the neuronal mechanism that governs insensitivity to time compression as much as three? And why does our tolerance to time-scale variability breaks down when the compression factor is greater than three?

Considering speech as an inherently rhythmic phenomenon, in which linguistic information is pseudo-rhythmically transmitted in syllabic packets^1^, Ghitza and Greenberg (2009) questioned whether intelligibility is influenced by neuronal oscillations. They measured the intelligibility of time-compressed speech subjected to “repackaging”—a process of dividing a time-compressed speech into fragments, called packets, and delivering the packets in a prescribed rate. As expected, the intelligibility of speech time-compressed by a factor of three (i.e., a high syllabic rate) was poor. Surprisingly, intelligibility was substantially restored when the information stream was re-packaged by inserting gaps in between successive compressed-signal intervals.

Conventional models of speech perception assume a strict decoding of the acoustic signal by linking time–frequency features of sensory input with stored time–frequency memory patterns. The intricate pattern of human performance as a function of speech speed and repackaging (i.e., the insensitivity to moderate time scale variations; the deterioration in intelligibility for compression factors beyond three; and the U-shaped recovery of intelligibility by repackaging) is difficult to explain by these models, but it can be accounted for by *Tempo* (Ghitza, 2011), a phenomenological model which epitomizes recently proposed oscillation-based models of speech perception (e.g., Poeppel, 2003; Ahissar and Ahissar, 2005; Lakatos et al., 2005; Ding and Simon, 2009; Ghitza and Greenberg, 2009; Giraud and Poeppel, 2012; Peelle and Davis, 2012). Tempo hypothesizes that the speech decoding process is performed within a time-varying, hierarchical window structure synchronized with the input. The window structure is generated by a cascade of oscillations with theta as “master,” capable of tracking the input pseudo-rhythm. During a successful tracking, the theta cycles are aligned with intervocalic speech fragments termed θ-syllables^2^. Oscillation-based models hypothesize that intelligibility is correlated with the ability of the theta oscillator to remain in sync with the input stream (e.g., Ghitza, 2012; Doelling et al., 2014). Intelligibility remains high as long as theta is in sync with the input (this is the case for moderate speech speeds) and sharply deteriorates once theta is out of sync (when the input syllabic rate is beyond the theta frequency range). Since the knee-point of intelligibility restoration defines the maximum *reliable* information transfer rate through the auditory channel (i.e., auditory channel capacity), one may conclude that the tracking capability of theta determines channel capacity. Can this conclusion account for the improvement in intelligibility gained by repackaging?

In interpreting the left-hand-side of their U-shaped behavioral data (i.e., increased intelligibility restoration with the increase of gap duration) Ghitza and Greenberg suggested that the insertion of gaps is an act of providing extra decoding time, and that the gradual change in gap duration should be viewed as tuning the packaging rate in a search for a better synchronization between the input information flow and the capacity of the auditory channel; repackaging with a gap duration (i.e., decoding time) that is too short results in errors due to a mismatch between the amount of information in the input stream (in terms of the number of diphones per unit time) and the capacity of the auditory channel (in terms of the number of reliable diphone-neuron activations per unit time). Consequently, they *hypothesized* that the optimal range of packaging rate is dictated by the properties of the cortical theta, and that the best synchronization is achieved by tuning the packaging rate toward the mid range of theta (Ghitza, 2011). Ghitza and Greenberg measured intelligibility as a function of gap duration (read: packaging rate) at only one time-compression condition (compression factor of three) and one packet duration condition (duration of 40 ms), with the operating points below capacity. In the study described here, we measured the knee-point of intelligibility restoration as a function of repackaging (with package duration and packaging rate as parameters) for fast speech with compression factors of up to eight. The combination of packaging rate and packet duration at knee-point defines the maximum rate at which speech information can be reliably transmitted through the auditory channel, i.e., the auditory channel capacity. As we shall see, irrespective of speech speed, the packaging rate and packet duration at capacity are aligned with properties of cortical theta, suggesting that the auditory channel capacity for speech is determined by theta.

The remainder of the paper is organized as follows. The psychophysical procedure to measure auditory channel capacity is described in section “Psychophysical measurement of auditory channel capacity.” Section “Material and methods” describes the speech corpus, the psychophysical paradigm, and the data analysis procedure; it also introduces definitions which will assist us in characterizing the relationship between the rate by which speech information is delivered to the listener, on the one hand, and intelligibility (i.e., a measure of the accuracy of speech perception), on the other. Three experiments are reported, in which intelligibility (in terms of word accuracy) is measured as a function of compression factor, packaging rate and packet duration. The stimulus preparation and the collected data, per experiment, are described in section “Results.” In section “Discussion” the data is interpreted through the prism of oscillation based models, and the possible generalizability of the results to other corpora (e.g., languages other than English) is discussed.

## 2. Psychophysical measurement of auditory channel capacity

**Figure 1:**
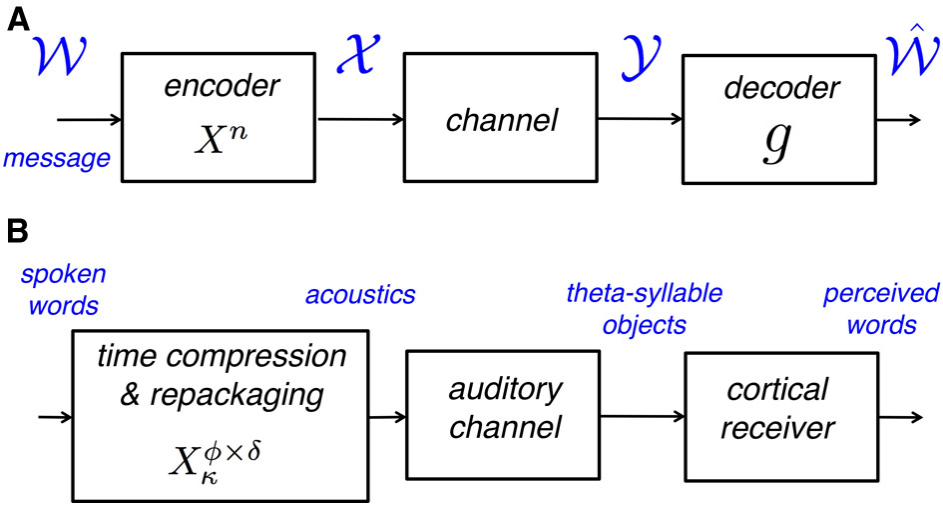
**(A)** A block diagram of a generic communication system. The encoder maps the source onto (binary) non-confusable, widely spaced input sequences to the noisy channel, so that a message can be transmitted with a desirably low probability of error. The maximum rate at which this can be done is called the *capacity of the channel*. **(B)** A block diagram of the auditory analog to the communication system in **(A)**. The encoder maps words onto acoustic waveforms and is defined by the time-compression factor, κ, and the parameters of the repackaging process, i.e., the packaging rate ϕ and the packet duration δ (see Figure 2). The channel is the auditory channel and the decoder is the cortical receiver, both defined in section “Psychophysical measurement of auditory channel capacity.”

Figure 1A shows a generic communication system for the transmission of a message that belongs to a set 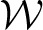 through a noisy channel. The system is composed of an encoder *X^n^*, the noisy channel, and a decoder *g*. The encoder maps messages 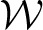 onto (binary) input sequences of length *n*, 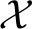, to the channel. The decoder maps the output sequences 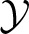 onto received-messages 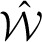. We seek encoders that produce a non-confusable, widely spaced input sequences to the channel. The highest *rate*, in bits per channel use, at which information can be sent with arbitrary low probability of error is called *channel capacity*. The encoders at capacity, *X*^n*^, satisfy $\mathrm{Pr}{\left\{\text{error}\right\}}_{\vec{X^{n}}}0$, or equivalently, $d_{\text{hamm}}{\left({\text{x}}_{i},{\text{y}}_{i}\right)}_{\vec{X^{n}}}0$ (measured at the decoder), where *d*_hamm_ is the *Hamming distance*^3^, and *x_i_, y_i_* are the input and output sequences, respectively.

To measure *auditory* channel capacity we translated the classic derivation (e.g., Shannon, 1948) into a psychophysical procedure. The auditory analog to the communication system in Figure 1A is shown in Figure 1B. The auditory channel is defined as follows:

Definition: The *auditory channel* includes all pre-lexical layers, with acoustic waveforms as input and syllable objects as output.

Corollary: The first layer of the *cortical receiver* is the lexical-access circuitry (i.e., words as output).

Such a partitioning of the auditory system stems from the postulation that, when engaging in a spoken dialog, the smallest linguistic meaningful units are words (e.g., Cutler, 1994, 2012).

**Figure 2:**
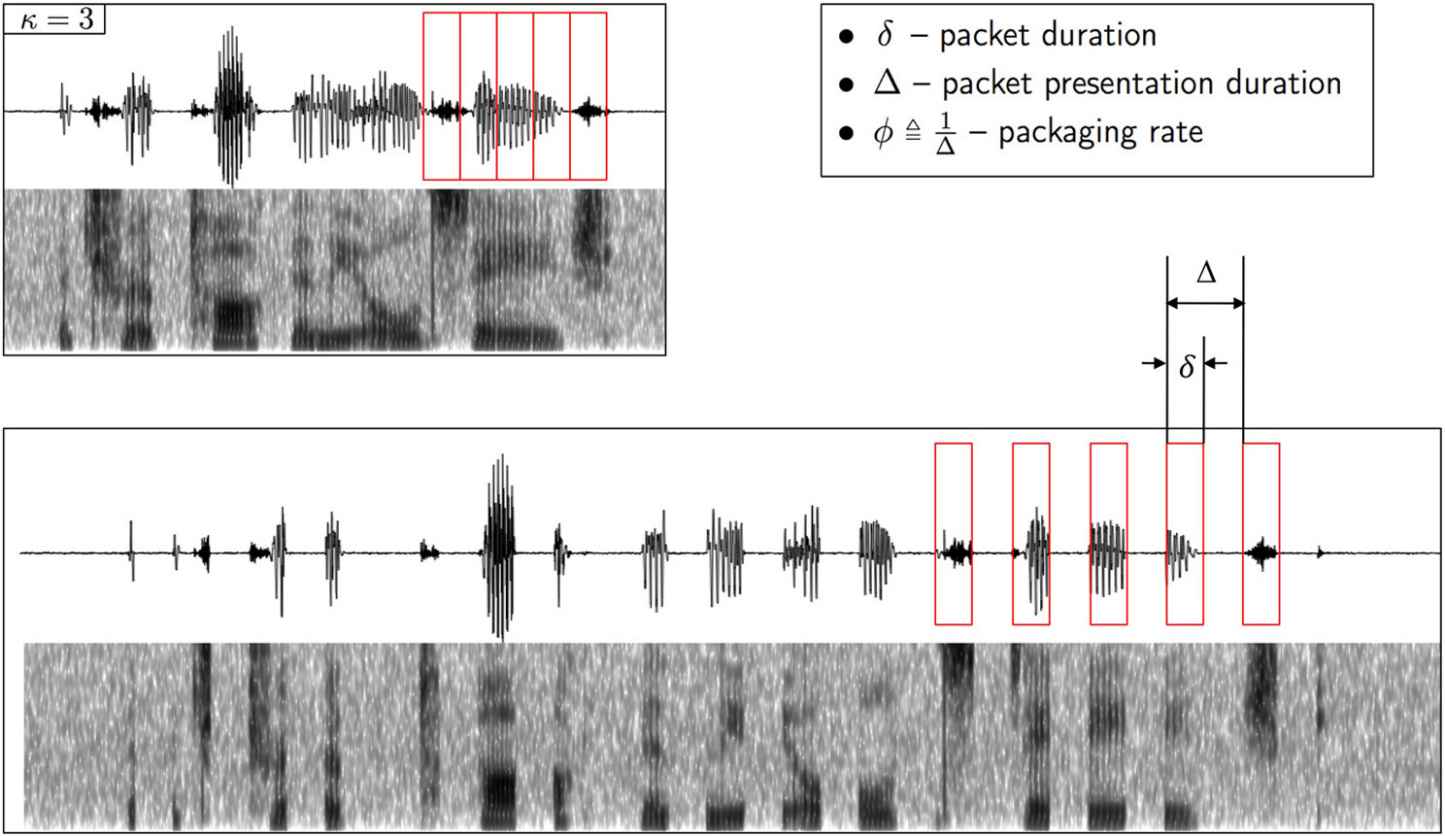
**Illustration of *repackaging* of a time compressed waveform**. The upper panel shows the waveform and the spectrogram of a sentence time compressed by a factor of κ = 3. The compressed waveform is blindly segmented into packets with equal duration of δ (red boxes, upper panel). The lower panel shows the time-compressed waveform after repackaging, with a packaging rate of $\phi \;≜\frac{1}{\Delta }$. The acoustic signal inside the δ-long packet is the time-compressed signal. A low-level background, speech-shaped noise is added (with SNR = 20 dB).

In the psychophysical realization, the encoding scheme is realized by a uniform time-compression operator, defined by the compression factor κ, followed by repackaging. Repackaging is defined by two parameters, the packaging rate ϕ and the packet duration δ (see Figure 2). The encoder is denoted *X*^ϕ × δ^_κ_: the subscript κ is the compression factor, and the superscript ϕ × δ defines the parameter space in the search for maximum intelligibility. The parameter values at optimum, κ, ϕ^*^ and δ^*^, define the encoder at capacity X^*^_κ_—the most favorable for the auditory channel; ϕ^*^ and δ^*^ define the maximum information transfer rate, hence enabling a quantitative estimate of auditory capacity. Since intelligibility is measured in terms of word accuracy, the search for optimal intelligibility restoration can be viewed as an act of minimizing *D*, *D* = *d*_hamm_ (w*_i_*, ${\hat{\text{w}}}_{i}$), where w*_i_*, ${\hat{\text{w}}}_{i}$ are the spoken and perceived *words*, respectively. D is defined at the receiver, in compliance with our way of partitioning the auditory system where the first layer of the cortical receiver is assumed to spell words as output. We assume that the cortical receiver is error free: as described in section “Material and Methods,” the behavioral task is a digit-string recognition, with a memory load of 4 digits. Such memory load is less than the immediate memory span, and the duration of 4 digits is less than the memory decay time (≅2 s, e.g., Cowan, 1984). Note that the assumption of an error free cortical receiver implies that errors are the result of erroneous representation of pre-lexical units, transmitted in a rate beyond capacity (i.e., errors are induced by the auditory channel).

## 3. Materials and methods

### 3.1. Subjects

All listeners, eight in number, were young adults (four female and four male college students, between 20 and 25 years of age) educated in the U.S.A. (English as first language) with normal hearing (screened for normal threshold audiograms). Their responses were reasonably consistent with each other, hence no further recruitment was needed.

### 3.2. Corpus

The experimental corpus comprised 100 digit strings spoken fluently by a male speaker. Each string is a 7-digit sequence, approximately 2 s long. It is uttered as a phone number in an American accent, i.e., a cluster of 3 digits followed by a cluster of 4 digits (for example: “two six two, seven zero one eight”). It is a low perplexity corpus (a vocabulary of 11 words, 0 to 9 and O) but semantically unpredictable. Each waveform file is accompanied by a phonetic transcription file, which includes the time instances of all acoustic landmarks including, in particular, vocalic nuclei (i.e., mid vowel markers^4^). These were marked by experienced phoneticians (by hand). For each signal condition, 80 stimuli (out of 100) were chosen at random and concatenated in a sequence: [alert tone] [digit string] [5-s long silence gap] [alert tone] …

### 3.3. Experimental paradigm

Subjects performed the experiment in an isolated office environment (no other occupants) using headphones. The sound pressure was adjusted by the subject to a comfort level and remained unchanged throughout the experiment. Stimuli were presented diotically. Each subject was tested on 50 signal conditions overall in 10 2-h sessions (5 conditions per session). Each condition was presented once, and the order of presentation was the same for all subjects. A condition comprised two phases, Training and Testing. The training set and the testing set contained 10 and 80 digit strings each, respectively, approximately 10 min to complete. Training preceded testing; in the training phase, subjects had to perform above a prescribed threshold before proceeding to the testing phase. Subjects were instructed to listen to a digit string once and, during the 5-s long gap following the stimulus, to type into an electronic file the last 4 digits heard, in the order presented (always 4 digits, even those that she/he was uncertain about). The rational behind choosing the last 4 digits as target (as opposed to choosing the entire 7-digit string) was two fold. First, it was an attempt to provide the opportunity for the presumed (cortical) theta oscillator to entrain to the input rhythm prior to the occurrence of the target words (recall the inherent rhythm in the stimuli, being a 7-digit phone number uttered in an American accent). Second, it aimed at reducing the bias of memory load on the error patterns.

The human-subjects protocol for this study was approved by the Institutional Review Board of Boston University. A participant provided hers/his written informed consent to participate in this study. This consent procedure was approved by the Institutional Review Board of Boston University.

### 3.4. Data analysis

The digit-string comprehension accuracy was measured as follows. Per stimulus, digit-string comprehension was define as *string correct C_i_*, with *C_i_* = 1 when the last 4 digits—as a whole—are correctly understood, and 0 otherwise. Per experiment, the data comprises 8 subjects, each of which was tested under *N* conditions, ψ ∈ {1, 2, …, *N*}, with 80 sentences heard under each condition (For example, in Experiment I, ψ is the compression factor κ, κ ∈ {2, 3, 4, 5}, i.e., *N* = 4). A hierarchical logistic regression was used to model the data, capturing the effect of each subject and each condition ψ on digit string comprehension. This approach is conceptually similar to a classical ANOVA comparison (Gelman, 2005): (a) inferences for all means and variances are performed under a model with a separate batch of effects for each row of the ANOVA table; (b) the model automatically gives the correct comparisons even in complex scenarios; and (c) this is a preferred approach when dealing with small sample size, as is the case here with only 8 subjects.

The model provides estimates for the average accuracy at each level of ψ. Instead of simply reporting standard errors for significance testing, this approach allows the flexibility of fully propagating the uncertainty inherent in all pieces of the model (Gelman and Hill, 2007). Here, this was done through a simulation framework, where the models estimates were simulated 1000 times. We computed 95% credible intervals around the accuracy levels at each ψ—these are the Bayesian equivalent of confidence intervals, again accounting for the full uncertainty in the model^5^.

The results plotted are estimates of percent correct, shown for each ψ, with error bars indicating the 95% credible intervals. Visually, we emphasize the credible interval around the estimated accuracy of ψ^*^—the reference condition. The estimated accuracy of the surrounding conditions are compared to the estimated accuracy of the reference condition, and the error bars indicate whether the differences are statistically significant when considering the credible intervals.

### 3.5. Definitions

Three quantities are defined, which will assist us in characterizing the relationship between the rate by which speech information is delivered to the listener, on the one hand, and intelligibility (i.e., a measure of the accuracy of speech perception), on the other. The first quantity is the *Articulated Speech Information (ASI)*, a measure of the amount of information carried by a fragment of time-compressed speech. The second quantity is the *ASI-Rate*—the rate by which the ASI is delivered. These measures characterize *stimulus properties* and have nothing to do with perception. The third quantity is the θ-*syllable*, an acoustic correlate of a unit of speech information defined by cortical function.

#### 3.5.1. Articulated speech information (ASI and ASIτ)

Since listeners are presented with time-compressed versions of the original waveform, a question arises: how to quantify the amount of information carried by a fragment of a *time-compressed* speech? For example, what is the amount of information within a 40-ms long interval of speech, time-compressed by a factor of 4? We propose to measure this quantity in terms of the information that *was intended* to be conveyed by the speaker when uttered (i.e., before compression).

Definition: the *Articulated Speech Information (ASI)*, denoted π, carried by a δ-long fragment of a κ-compressed stimulus is the amount of information, in bits, in the corresponding uncompressed fragment.

Note that the speech fragment in question is arbitrary, i.e., it doesn't have to be aligned with any particular linguistic unit.

**Figure 3:**
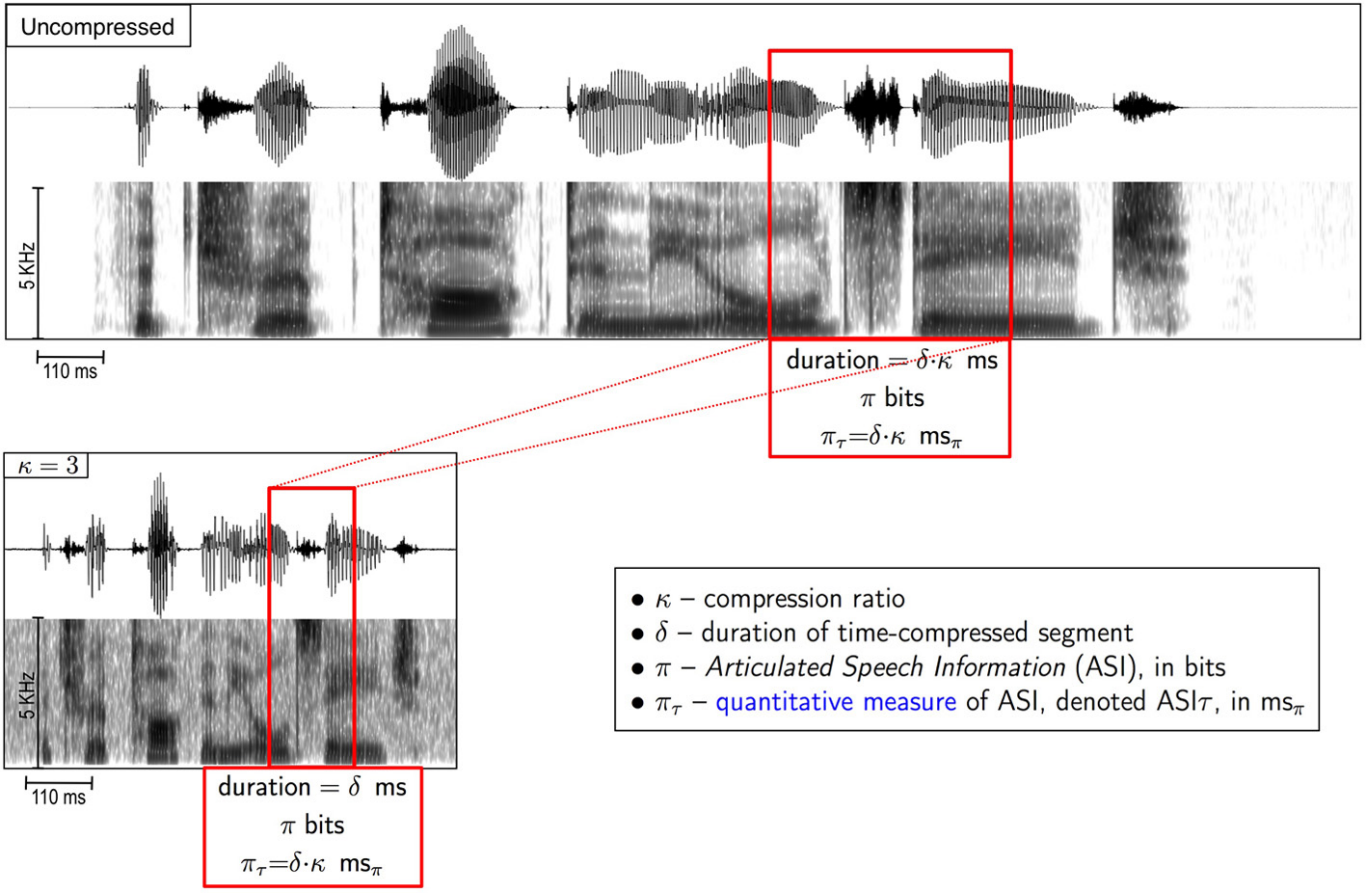
**What is the amount of speech information carried by a fragment of a time-compressed speech?** We define *Articulated Speech Information* (ASI) carried by a δ-long segment of a κ-compressed stimulus (red box in lower panel) as the amount of information, in bits, in the corresponding uncompressed segment (red box, upper panel). ASI is the speech information that was intended to be conveyed by the speaker when uttered (i.e., before compression). See text (section “Definitions”) for the definition of ASIτ.

In our study a speech corpus with low perplexity is used (7-digit strings). In this case, it is reasonable to assume that the ASI carried by a speech fragment that is a few tens of milliseconds long is related to the *duration* of the uncompressed fragment, i.e., π ~ δ·κ (see Figure 3).

Definition: *ASI*τ, denoted π_τ_, is an *estimate*—in time units—of the ASI carried by a δ-long fragment of a κ-compressed stimulus, equals δ·κ. To distinguish duration (of a time-interval) from ASIτ—both measured in time units—we denote 1 ms of ASIτ as 1 ms_π_.

That is, for the 7-digit strings corpus we assume {ASI, in bits} ~ {ASIτ, in ms_π_}. In our example, the ASI (π, in bits) carried by a 40-ms long fragment of speech time-compressed by 4 is related to an ASIτ that equals π_τ_ = 40 · 4 = 160 ms_π_.

It is worth emphasizing that there is a distinction between ASI, the amount of information *articulated* by the speaker (i.e., intended to be conveyed), and the amount of information *perceived* by the listener. During the decoding process some of the articulated information may be lost; the amount of the loss depends on κ and is measured with respect to the ASI.

#### 3.5.2. ASI-Rate and ASIτ-Rate

Let *ASI-Rate*—or, equivalently, *ASIτ-Rate*—be the *information rate* in transmitting π bits of ASI—or, equivalently, π_τ_ ms_π_ of ASIτ—by a δ-long fragment of κ-compressed speech, and let both be denoted *R*^κ^_δ_. Then:

$$R_{\delta }^{\kappa }=\frac{\pi }{\delta }\text{ bits}/\text{s}\sim \frac{\pi_{\tau }}{\delta }\;{\text{ms}}_{\pi }\text{/s}$$

In the reminder of the paper we shall omit, for simplicity, the subscript and superscript of *R*^κ^_δ_ using *R* instead, measured in ms_π_/s.

#### 3.5.3. The θ-syllable

A widely accepted assessment is that a consistent acoustic correlate to the (conventional) syllable is hard to define (e.g., Cummins, 2012). Concurring with this assessment, and in light of the proposed role of the theta oscillator in governing the decoding process (e.g., Ghitza, 2011; Giraud and Poeppel, 2012), Ghitza (2013) suggested the θ-syllable as an alternative unit, inspired by brain function:

Definition: A θ-syllable is a θ-cycle long speech segment located in between two successive vocalic nuclei.

During a successful tracking by the theta oscillator (for uncompressed speech, in quiet, this is the normative case) one θ-cycle is aligned with the interval between two successive vocalic nuclei. As such, the θ-syllable is a non-ambiguous acoustic correlate to a VΣV (the Σ stands for *consonant cluster*). Given the prominence of vocalic nuclei in the presence of environmental noise, the θ-syllable is robustly defined. The θ-syllable is also invariant to time scale modifications that result in intelligible speech. When listening to time-compressed speech that is intelligible, the cortical theta is in sync with the stimulus. Thus, the speech fragment that corresponds to a theta cycle is the time-compressed version of the corresponding uncompressed VΣV fragment (Ghitza, 2013).

## 4. Results

### 4.1. Overview

Three experiments were conducted. In Experiment I, listeners were presented with time-compressed speech *without* repackaging, with the time-compression factor, κ, the parameter. Speech information is delivered in a “natural way,” i.e., the “packaging rate” is the syllabic rate of the stimulus and a packet is the time-compressed θ-syllable. The goal is to find κ^*^, the κ at knee-point of performance. The θ-syllable rate at knee point is denoted ϕ^*^, and the average “packet presentation” duration is the duration of a ϕ^*^ cycle, $\Delta^{*}=\frac{1}{\phi^{*}}$. In Experiment II, κ is increased beyond κ^*^, resulting in a deterioration in performance. Intelligibility is recovered by launching the repackaging process depicted in Figure 2, with a parameter search in the ϕ × δ space (i.e., the [packaging-rate]×[packet-duration] space). The parameter values at optimum, ϕ^*o*^ and δ^*o*^, define the information rate at the optimal recovery point, denoted *R*^*o*^^6^. This process is repeated for every value of κ, κ > κ^*^; as we shall see, *R*^*o*^ is independent of κ. In Experiment III, we verify that *R*^*o*^ is indeed an estimate of the auditory channel capacity.

### 4.2. Experiment I: increase κ to knee-point of performance

#### 4.2.1. Stimulus preparation

The compression factor, κ, was gradually increased to a knee-point of performance, measured in terms of word recognition accuracy. The waveforms were time-compressed using a pitch-synchronous, overlap and add (PSOLA) procedure (Moulines and Charpentier, 1990) incorporated into PRAAT, a speech analysis and modification package (http://www.fon.hum.uva.nl/praat/). The formant patterns and other spectral properties of the time-compressed signal are preserved but altered in duration (compare upper and lower panels in Figure 3), however, the fundamental frequency (“pitch”) contour remains the same^7^. Note that, by definition, the ASIτ within a κ-compressed θ-syllable (i.e., an intervocalic segment, κ-compressed) is same for all κ, equals to π_τ_ ms_π_.

Let κ at knee-point be denoted κ^*^. We define:

(1) $$\phi^{*}\;≜E\;\left\{\frac{1}{T_{V\Sigma V}^{*}}\right\}$$

(2) $$\Delta^{*}=\frac{1}{\phi^{*}}$$

(3) $$\pi_{\tau }^{*}=\Delta^{*}\cdot \kappa^{*}$$

(4) $$R^{*}=\frac{\pi_{\tau }^{*}}{\Delta^{*}}$$

T^*^_VΣ*V*_ is the duration of an intervocalic segment at κ^*^ (equals the difference between two successive vocalic nuclei marked as described in subsection “Corpus”), ϕ^*^ is the average natural packaging rate of the κ^*^-compressed waveform, Δ^*^ is the average packet presentation duration, and π^*^_τ_ and R^*^ are the average ASIτ and the average ASIτ-Rate at knee-point, respectively. The drop in performance for κ > κ^*^ is *interpreted* to be the result of the cortical θ reaching the upper limit of its frequency range, θ_max_ (Ghitza, 2011). A corollary to this interpretation is that ϕ^*^ reflects θ_max_. Note that, biophysically, θ_max_ is not a cutoff frequency in a “brick-wall” sense; rather, θ diminishes in a gradual manner. In the reminder of the paper we shall assume a brick-wall θ_max_.

#### 4.2.2. Data

**Figure 4:**
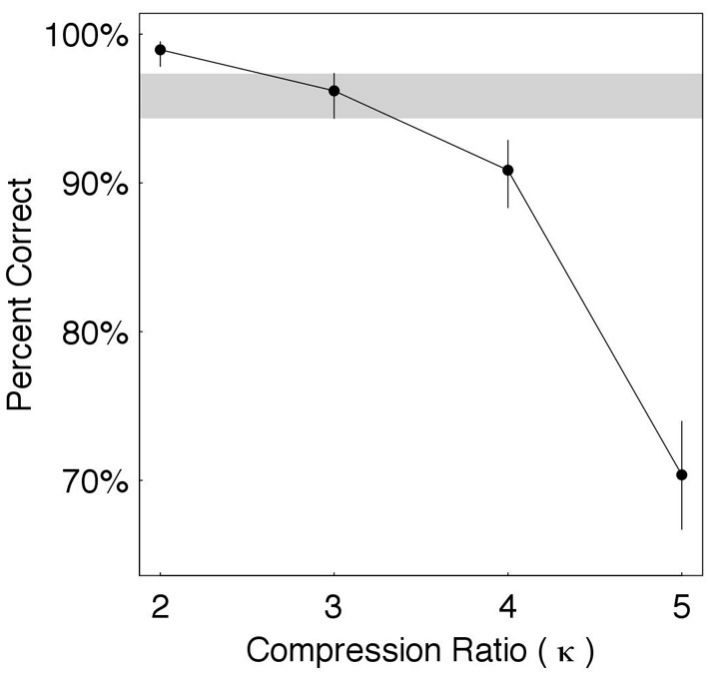
**Time compression without gaps**. Shown are estimates of word recognition accuracy (in percent correct) for each κ ∈ {2, 3, 4, 5}, with error bars indicating the 95% credible intervals. The knee-point is at κ^*^ = 3, with the credible interval around it visually highlighted (gray horizontal strip).

The results are shown in Figure 4. Estimates of word recognition accuracy (in percent correct) are shown for each κ ∈ {2, 3, 4, 5}, with error bars indicating the 95% credible intervals. To determine the knee-point of performance we compare the estimated accuracy at a prescribed candidate condition with the accuracy at the preceding and following conditions. Shown is a candidate condition κ = 3, with the credible interval around it visually highlighted (gray horizontal strip). The estimated accuracy at κ = 3 is 96%—quite close to 99% (average accuracy when κ = 2) and considerably better than 91% (when κ = 4). The error bars indicate that, in both cases, the differences are statistically significant when considering the credible intervals. Consequently, the knee-point is determined to be κ^*^ = 3.

Using Equations (1)–(4) we obtain that at κ^*^ = 3: ϕ^*^ = 9 Hz, Δ^*^ = 110 ms, π^*^_τ_ = 330 ms_π_, and $R^{*}=\frac{\pi_{\tau }^{*}}{\Delta^{*}}=\frac{330}{110}=3\;{\text{ms}}_{\pi }\text{/ms}$. In words, at knee-point, the average packaging rate is 9 θ-syllables/s, a packet is a κ^*^-compressed θ-syllable with an average duration of 110 ms, the ASIτ carried by a packet is the duration of an uncompressed θ-syllable with an average duration of 330 ms_π_, and the information transfer rate is 3 ms of ASIτ (measured in ms_π_) per 1 ms of time-compressed waveform.

### 4.3. Experiment II: increase κ beyond knee-point

#### 4.3.1. Stimulus preparation

The compression factor, κ, was increased beyond κ^*^, resulting in a massive deterioration in performance (see, for example, performance at κ = 5, shown in Figure 4). To recover performance repackaging was applied. In accordance with the interpretation that ϕ^*^ reflects θ_max_ (subsection “Experiment I”) packaging rate was frozen at ϕ^*^ for all values of κ, κ > 3, leaving the packet duration, δ, as the only varying parameter in the search for optimal recovery. Packet duration at knee-point of optimal recovery is denoted δ^*o*^, and the ASIτ carried by this packet is:

(5) $$\pi_{\tau }^{o}=\delta^{o}\cdot \kappa$$

hence the ASIτ-Rate:

(6) $$R^{o}=\frac{\pi_{\tau }^{o}}{\Delta^{*}}$$

We seek *R^o^* (the ASIτ-Rate at optimal recovery) as a function of κ. Since Δ^*^ is same for all κ (because ϕ^*^ is frozen), seeking *R*^*o*^ is equivalent to seeking π^*o*^_τ_ [the ASIτ at optimal recovery, see Equation (6)].

#### 4.3.2. Data

*R*^*o*^ was measured for κ ∈ {4, 5, 6, 7, 8}. For each κ, packaging rate was frozen at ϕ^*^ = 9 Hz (with Δ^*^ = 110 ms), and packet duration δ was the search parameter. Five values of δ were used, defined by five prescribed values of ASIτ: π_τ_ = [230 280 **330** 380 430] ms_π_. (Note that the mid-value of the five-value π_τ_ is 330 ms_π_—the ASIτ at the knee-point κ^*^ = 3; see Experiment I.) Same five-value π_τ_ was used for all κ. For a given κ, δ was derived from π_τ_ as:

(7) $$\delta =\frac{\pi_{\tau }}{\kappa }\text{ ms}$$

For example, for κ = 5 Equation (7) yields δ = [46 56 66 76 86] ms. With packaging rate frozen at ϕ^*^ = 9 Hz, the five-value δ defines five repackaging conditions per κ.

**Figure 5:**
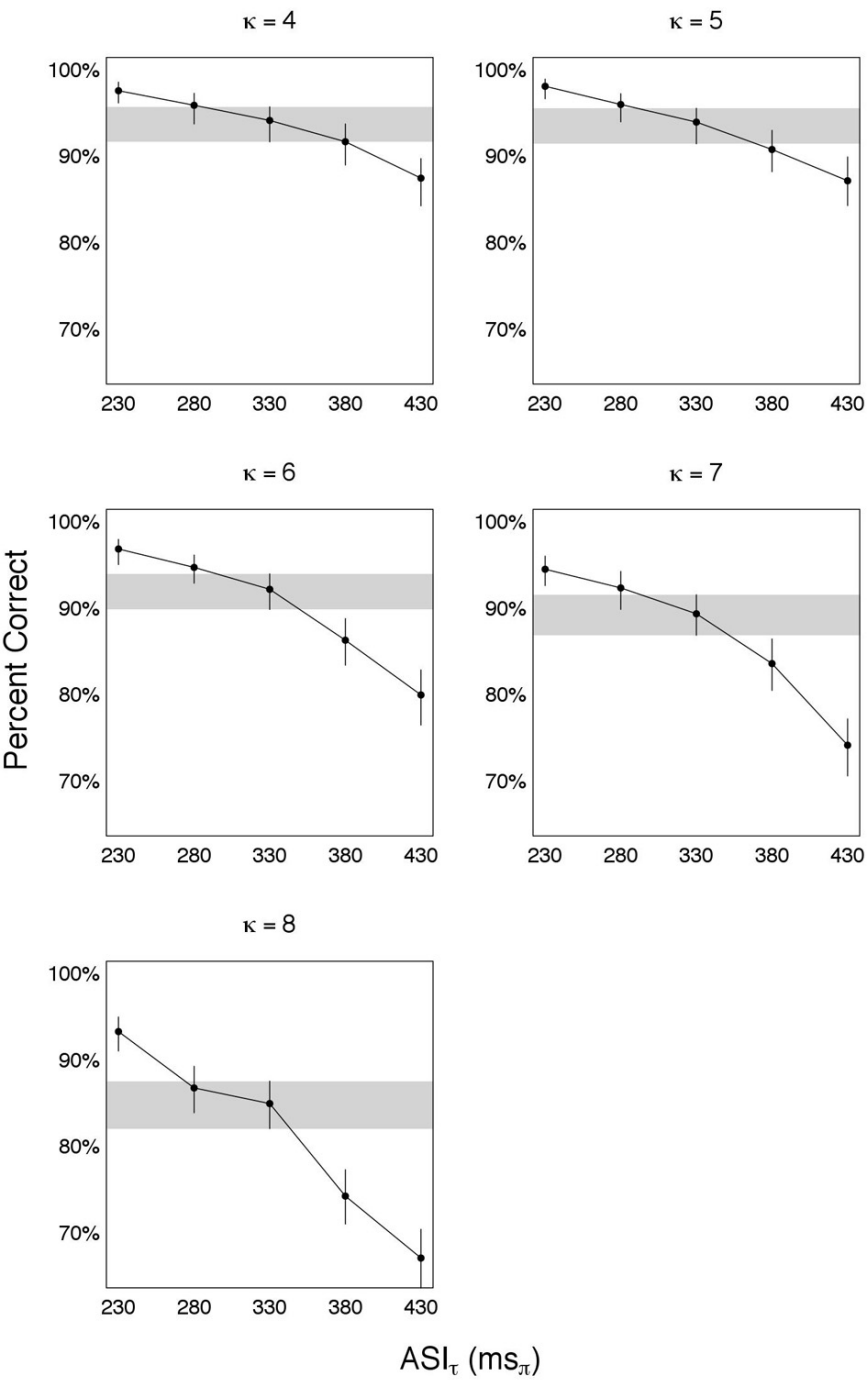
**Time compression with κ > 3**. Such degree of time compression results in a massive deterioration in performance. To recover performance repackaging was applied, with a packaging rate of ϕ^*^ = 9 Hz. Five panels are shown, one for each κ. For each panel, estimates of accuracy (in percent correct) are shown for each π_τ_ ∈ {230, 280, 330, 380, 430} ms_π_, with error bars indicating the 95% credible intervals. The knee-point of recovery is at 330 ms_π_, with the credible interval around it visually highlighted (gray horizontal strip). ASIτ at knee-point is a *constant*, independent of κ, equals the average duration of one uncompressed θ-syllable and delivered in κ-compressed θ-syllable long packets. Since the packaging rate ϕ^*^ = 9 Hz (interpreted to be equal to cortical θ_max_), the information transfer rate at knee-point of recovery is 9 θ-syllables/s.

The results—shown in Figure 5—are organized in five panels, one for each κ ∈ {4, 5, 6, 7, 8}. For each panel, estimates of accuracy (in percent correct) are shown for each π_τ_ ∈ {230, 280, 330, 380, 430} ms_π_, with error bars indicating the 95% credible intervals. To determine the knee-point of performance we compare the estimated accuracy at a prescribed candidate condition with the accuracy at the preceding and following conditions. Shown is a candidate condition π_τ_ = 330 ms_π_, with the credible interval around it visually highlighted (gray horizontal strip). The estimated accuracy at π_τ_ = 330 ms_π_ is quite close to the accuracy at π_τ_ = 280 ms_π_, and considerably better than the accuracy at π_τ_ = 380 ms_π_ (this is especially so for κ = 6, 7, and 8). The error bars indicate that the differences in estimated accuracies are statistically significant when considering the credible intervals. Consequently, the knee-point is determined to be at π^*o*^_τ_ = 330 ms_π_. Relating this finding to the finding of Experiment I reveal:

(8) $$\pi_{\tau }^{o}≅\pi_{\tau }^{*}={\text{330 ms}}_{\pi },\;\forall \kappa$$

That is, ASIτ at knee-point of recovery is a *constant*, independent of κ, equals the average duration of one uncompressed θ-syllable and delivered in κ-compressed θ-syllable long packets. Since the packaging rate is ϕ^*^ = 9 Hz (interpreted to be equal to cortical θ_max_), the information transfer rate at knee-point of recovery is 9 θ-syllables/s. Or, expressed in ASIτ-Rate:

(9) $$R^{o}=\frac{\pi_{\tau }^{o}}{\Delta^{*}}=\frac{\pi_{\tau }^{*}}{\Delta^{*}}=R^{*}=3{\text{ms}}_{\pi }/\text{ms},\;\forall \kappa$$

That is, the ASIτ-Rate is a constant, equals to R^*^ = 3 ms_π_/ms, for all κ.

### 4.4. Experiment III: are we at capacity?

#### 4.4.1. Stimulus preparation

In Experiment II we found that the ASIτ-Rate at optimal recovery is *R*^*o*^ = R^*^ = 3 ms_π_/ms, for all κ's. The ϕ^*^ and δ^*o*^ combination that determined *R*^*o*^ was ϕ^*^ = 9 Hz and δ^*o*^—the duration of a κ-compressed speech fragment with ASIτ π^*o*^_τ_ = 330 ms_π_. For *R*^*o*^ to be considered capacity we must show that there exist no *R* > *R*^*o*^ which maintains performance. In the experiment described here we measured performance for *R*'s with *R* > *R*^*o*^, and found that performance deteriorated for all *R*'s tested, thus concluding that *R*^*o*^ is indeed an estimate of auditory capacity.

#### 4.4.2. Data

**Figure 6:**
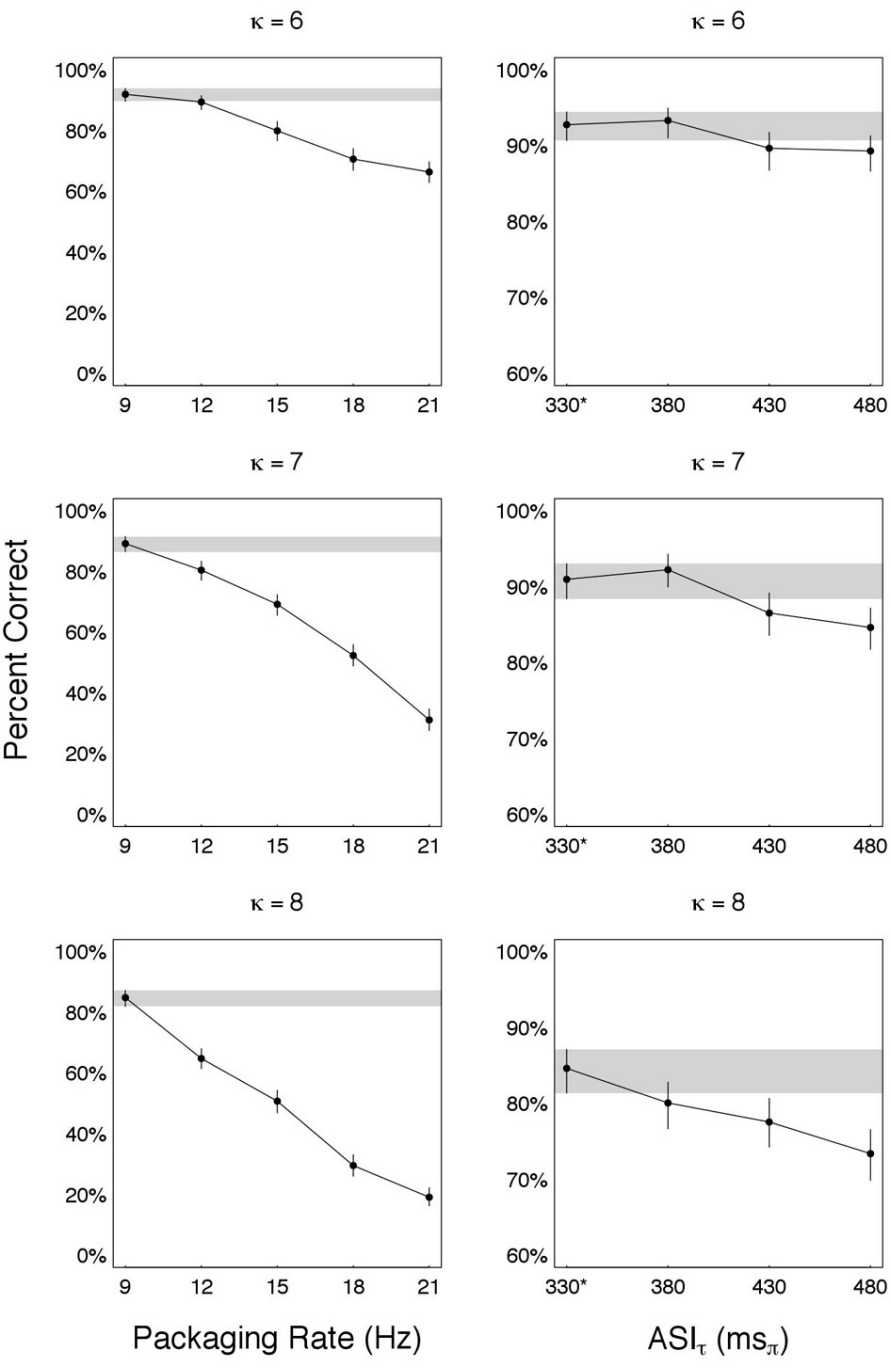
**Are we at capacity?** Performance for combinations of packaging-rate×packet-duration with information rates *R* greater than *R*^*o*^—the rate at optimal recovery. (*Left*) Estimated accuracy as a function of packaging rate ϕ > ϕ^*^ = 9 Hz. For all ϕ, packet duration is such that ASIτ is a constant (equals 330 ms_π_). The reference condition is at *R*^*o*^ (i.e., ϕ^*^ = 9 Hz and π^*o*^_τ_ = 330 ms_π_), with the credible interval around it visually highlighted (gray horizontal strip). (*Right*) word accuracy as a function of ASIτ > π^*o*^_τ_ = 330 ms_π_. Packaging rate was reduced to ϕ = 5 Hz in order to maintain a packet duration δ that is smaller than the packet presentation duration $\frac{1}{\phi }$. The reference condition is at *R*^*o*^, denoted 330^*^ in the figure (the star indicates ϕ^*^ = 9 Hz, as opposed to ϕ = 5 Hz in all other ASIτ values), with the credible interval around it visually highlighted (gray horizontal strip). In both, left and right columns, *R*^*o*^ gives the best performance ⇒ *R*^*o*^ is the auditory channel capacity, 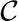
_auditory_ = 9 θ-syllables/s.

Recalling that $R^{o}=\frac{\pi_{\tau }^{o}}{\Delta^{*}}=\pi_{\tau }^{o}\cdot \phi^{*}$, we obtained *R* > *R*^*o*^ by using ϕ > ϕ^*^ while keeping π_τ_ = π^*o*^_τ_. In particular, we used π_τ_ = 330 ms_π_ and ϕ = [12 15 18 21] Hz ⇒ *R* = $\frac{1}{3}$ · ϕ = [4 5 6 7] ms_π_/ms (each entry greater than *R*^*o*^ = 3 ms_π_/ms). The results—shown in the left-hand-side column of Figure 6—are organized in three panels, one for each κ ∈ {6, 7, 8}. For each panel, estimates of accuracy (in percent correct) are shown for each ϕ ∈ {9, 12, 15, 18, 21} Hz, with error bars indicating the 95% credible intervals. The reference condition is at *R*^*o*^ (i.e., ϕ^*^ = 9 Hz and π^*o*^_τ_ = 330 ms_π_), with the credible interval around it visually highlighted (gray horizontal strip).

We also measured performance for π_τ_ > π^*o*^_τ_, i.e., a packet duration $\delta =\frac{\pi_{\tau }}{\kappa }>\delta^{o}=\frac{\pi_{\tau }^{o}}{\kappa }$, the duration at optimal recovery. We chose δ's defined by π_τ_ = [380 430 480] ms_π_. In order to maintain a packet duration δ that is smaller than the packet presentation duration $\frac{1}{\phi }$, packaging rate was reduced to ϕ = 5 Hz. Note that for such choice of ϕ, *R* = [1.9 2.15 2.4] ms_π_/ms (each entry smaller than *R*^*o*^ = 3 ms_π_/ms). The results—shown in the right-hand-side column of Figure 6—are organized in three panels, one for each κ ∈ {6, 7, 8}. For each panel, estimates of accuracy (in percent correct) are shown for each π_τ_ ∈ {330^*^, 380, 430, 480} ms_π_, with error bars indicating the 95% credible intervals. The reference condition is at *R*^*o*^, denoted 330^*^ in the figure (the star indicates ϕ^*^ = 9 Hz, as opposed to ϕ = 5 Hz in all other π_τ_ values), with the credible interval around it visually highlighted (gray horizontal strip).

In both tests *R*^*o*^ gives the best performance, leading to the conclusion that *R*^*o*^, indeed, is the auditory channel capacity, denoted 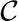
_auditory_.

## 5. Discussion

Conceptually, information transfer rate can be expressed in units of bits/s (ASI-Rate), ms_π_/s (ASIτ-Rate), or θ-syllables/s. As we shall see in subsection “How generalizable are our findings?,” θ-syllables/s is the most insightful unit.

**Figure 7:**
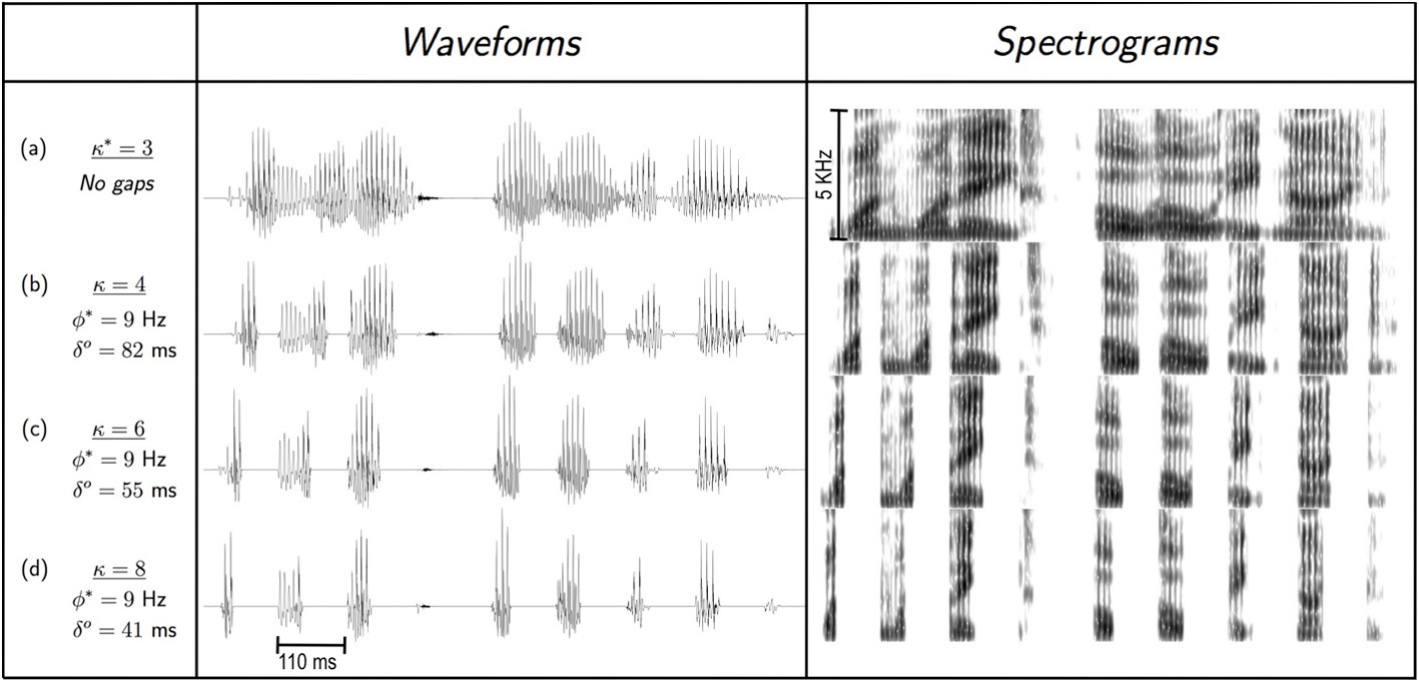
Packaging rate, ϕ^*^, and packet duration, δ^*o*^, at capacity. For uncompressed speech (i.e., κ = 1, not shown), speech information is delivered naturally: the packaging rate is the nominal syllabic rate (≅ 3 syllables/s, for our speech corpora) and a packet is a θ-syllable with an average duration of ≅ 330 ms. **(A)** Knee-point of performance for uniform time-compression *without* gaps, κ^*^ = 3. Speech information is delivered naturally, where the packaging rate, ϕ^*^, is the syllabic rate of the stimulus (≅ 9 syllables/s), in correspondence with the upper limit of theta, θ_max_ ≅ 9 Hz. The duration of a ϕ^*^ cycle—the packet presentation duration—is Δ^*^ = 1/ϕ^*^ ≅ 110 ms, and the average natural-packet duration is δ^*^ = Δ^*^ = 110 ms. **(B)** A uniform compression with κ = 4, which results in a deterioration in performance, is followed by repackaging to restore performance. Packaging rate is kept at ϕ^*^ ≅ 9 packets/s, hence Δ^*^ ≅ 110 ms. Packet duration at optimal restoration is the duration of a θ-syllable, time-compressed by κ = 4, i.e., δ^*o*^ = 330/4 = 82.5 ms. Entries in the remaining rows are derived in an analogous manner. Note that in rows **(B–D)** packets are delivered with an identical packaging rate, and the *articulated speech information*—in terms of time-frequency signature—carried by a particular packet in rows **(C,D)** is the same as in the corresponding packet in row **(B)**, although with different acoustic realization (due to different compression factor).

In Experiment I we found that for time compression without repackaging, knee-point of performance is at κ^*^ = 3. The “natural packaging” rate (i.e., the syllabic rate) is ϕ^*^ ≅ 9 natural-packets/s—in correspondence with θ_max_, the upper limit of cortical theta (≅9 Hz)—and one natural-packet contains one θ-syllable [Figure 7, row (**A**)]. Hence, the information transfer rate, in units of θ-syllables/s is:

$$R^{*}=9\;\theta \text{-syllables/s}$$

Since the corresponding ASIτ is π^*^_τ_ = 330 ms_π_, and the duration of a natural packet is $\delta^{*}=\Delta^{*}=\frac{1}{\phi^{*}}≅110\text{ ms}$, the information transfer rate in units of ms_π_/s is:

$$R^{*}=\frac{\pi_{\tau }^{*}}{\delta^{*}}=\frac{330}{110}=3\;{\text{ms}}_{\pi }/\text{ms}$$

In Experiment II we found that for all κ > 3, with packaging rate of ϕ^*o*^ = ϕ^*^ ≅ 9 packets/s, at knee-point of intelligibility recovery a packet carries an ASI of one θ-syllable long speech fragment. Hence, the information transfer rate in units of θ-syllables/s is:

$$R^{o}=9\;\theta \text{-syllables/s}=R^{*}\;\forall \kappa >3$$

The packet duration equals the duration of the θ-syllable compressed by κ [Figures 7, rows (**B–D**), and the corresponding ASIτ, π^*o*^_τ_ = 330 ms_π_, is delivered within a packet presentation duration of $\Delta^{o}=\Delta^{*}=\frac{1}{\phi^{*}}≅110\text{ ms}$. Therefore, the information transfer rate, in units of ms_π_/s is:

$$R^{o}=\frac{\pi_{\tau }^{o}}{\Delta^{*}}=\frac{330}{110}=3\;{\text{ms}}_{\pi }/\text{ms}=R^{*}\;\forall \kappa >3$$

Finally, in Experiment III we found that performance deteriorates for all *R*>*R*^*o*^ or π_τ_>π^*o*^_τ_ tested.

Based on these findings we conclude:

1. The auditory channel can reliably transmit, *at most*, the ASI in one θ-syllable long speech fragment per one θ_max_ cycle, independent of κ.
2. *R*^*o*^ is the auditory channel capacity, 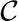
_auditory_. This is so because all other combinations of [packaging-rate] × [packet-duration] with higher bit rates result in higher error rates. Expressed in θ-syllables/s, 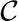
_auditory_ = 9 θ-syllables/s.
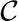
_auditory_ is determined by cortical θ. This is so because for all κ, at capacity, the maximum information reliably decoded is the ASI of one θ-syllable long speech fragment, delivered in κ-compressed θ–syllable long packets in a rate of ϕ ≅ 9 packets/s ≅ cortical θ_max_.

### 5.1. Relation to oscillation-based models

In accordance with our definition (see section “Psychophysical measurement of auditory channel capacity”), the auditory channel includes all pre-lexical layers (including Tempo), with acoustic waveforms as input and θ-syllable objects as output. Reiterating the cortical computation principle embodied in Tempo, the speech decoding process is performed within a hierarchical window structure synchronized with the input, generated by a cascade of oscillations capable of tracking the input pseudo-rhythm. Performance remains high as long as theta, the master, is in sync with the input, and sharply deteriorates once theta is out of sync.

Examining the findings of our study through the prism of Tempo, for time-compressed speech with κ < 3 and without repackaging, the syllabic rate is within the theta range. Synchronization is thus maintained and theta cycles are aligned with intervocalic acoustic segments (i.e., θ-syllables). For κ > 3 performance sharply deteriorates because the syllabic rate (now greater than 9 syllables/s) is outside the range of theta ⇒ theta is out of sync. Repackaging restores intelligibility. A revealing finding is that, *at capacity*, with a packaging rate of 9 packets/s (and synchronization now maintained), a packet contains the information in a speech fragment that is *one* uncompressed θ-syllable long, independent of κ (the duration of the packet equals one κ-compressed θ-syllable).

### 5.2. Synthesis by repackaging: acoustics vs. intelligibility

There is a distinction between the speech information carried by a stimulus and the speech information reliably perceived by the listener. The repackaged stimuli are assumed to contain all speech information articulated by the speaker (i.e., intended to be conveyed). (This assumption is based upon *objective* criteria, e.g., the ability to recover the uncompressed signal from the repackaged version.) During the human decoding process, however, some of this information is lost, and the extent of loss is quantified by measuring intelligibility. In this study, stimuli were defined by the repackaging parameters κ, ϕ, and δ, and capacity was defined as the knee-point of intelligibility recovery. What are the auditory functions responsible for the intelligibility loss when listening to repackaged stimuli, and how the synthesis parameters (which define the stimulus) and the auditory channel parameters interact? We shall use the Tempo model to examine this interaction.

According to Tempo, as long as ϕ is inside the cortical θ frequency range, the window structure is determined by ϕ (Ghitza, 2011): cortical θ is in sync with ϕ, and as the master in the cascaded oscillators array it determines β and γ (via cascading). The β cycles (entrained to θ) define the windows within which the phonetic content is decoded, and the decoding is via sampling the sensory information inside the β cycle in a γ pace (entrained to β); the sampling time-instances are in phase with the β cycle (see Appendix in Ghitza, 2011).

Two cases of stimulus vs. auditory parameter interaction are examined. First, as described in the “Stimulus preparation” subsection of Experiment I, the uniform time compression is in the PSOLA sense; i.e., only the vocal-tract movement is speeded up while the pitch contour remains unchanged. If the packet duration of a repackaged stimulus (δ) is smaller than one pitch-period the pitch contour is severely distorted, resulting in deterioration in intelligibility. For all stimuli used in our study, a packet lasted a few pitch periods (see, for example, Figure 7).

Second, the accuracy of decoding depends on the interaction between the stimulus parameters κ and δ, and the auditory parameter γ. In particular, if the duty cycle of the repackaged stimulus is two small (i.e., if δ is too short compared to the ϕ cycle), the γ-driven sampling may be too coarse (recall that γ is dictated by ϕ, via cascading). Undersampling will also occur if the signal inside the packet is overly compressed (κ is too large). These examples illustrate that, for a given ϕ, intelligibility is affected by the choice of κ and δ. Interestingly, our study shows that for all five repackaging conditions tested (i.e., κ ∈ {4, 5, 6, 7, 8}, all with ϕ = 9 Hz), capacity is reached for a δ that is a κ-compressed θ-syllable long speech fragment. The fact that, at capacity, both ϕ and δ correspond to cortical θ leads to the inference that auditory channel capacity is determine by cortical θ.

### 5.3. How generalizable are these findings?

Our estimate of auditory channel capacity, 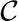
_auditory_, was measured for English digit strings spoken by a male talker speaking in a “nominal” rate. Will this estimate generalize to digit strings spoken by a “fast” talker? to English speech corpora with higher perplexity? to speech corpora in other languages?

In Shannon's framework, capacity is determined by the channel (Shannon, 1948). Note that the *auditory* channel as we define it (see section “Psychophysical measurement of auditory channel capacity”) is a *time-varying* channel: because it operates within a window structure synchronized with the input rhythm, the auditory channel is a function of the input, hence time-dependent. Nevertheless, at capacity the channel can be assumed stationary because the window structure is frozen as the master window is determined by θ_max_. With this observation in mind, we suggest the following predictions:

1. *A 7-digit strings corpus spoken by “fast” talkers*. At capacity, packaging rate ϕ^*^ = 9 packets/s, interpreted to be determined by θ_max_ = 9 Hz. If we assume same θ_max_ across gender and race (indeed species; e.g., Buzsaki et al., 2013), in a repeat of Experiment I, κ at knee-point of performance (κ^*^_fast_) should be such that ϕ^*^ = θ_max_, with π^*^, π^*^_τ_ and R^*^ as measured for the male talker. Since the syllabic rate for a fast talker is higher than the syllabic rate of a male talker, we expect κ^*^_fast_ < κ^*^ = 3. In a repeat of Experiment II (now κ > κ^*^_fast_) the search for optimal recovery of intelligibility should yield δ^*o*^, π^*o*^, π^*o*^_τ_ and *R*^*o*^ as measured for the male talker (as dictated by θ_max_). We therefore predict that 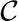
_auditory_—estimated for 7-digit strings spoken by a male talker—will generalize, in θ-syllables/s, bits/s or ms_π_/s units.
2. *English speech corpora with higher perplexity*. Using a rational similar to the one used for fast talkers, in a repeat of Experiment I, κ at knee-point of performance should be such that ϕ^*^ = θ_max_, with a distribution of *compressed* θ-syllable durations similar to that of a compressed English digit-string source. However, the average ASI (in bits) carried by a θ-syllable in a corpora with a higher perplexity would be greater than that of the English digit-string corpus (because of the reacher VΣV inventory). It is therefore predicted that, expressed in θ-syllables/s, capacity will generalize (to be 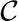
_auditory_ = 9 θ-syllables/s); however, if expressed in bits/s, the auditory channel capacity for English speech corpora will be greater than that for a 7-digit strings corpus (with lower perplexity). Measuring capacity in ms_π_/s units is inapplicable here because the relationship at the core of the ASIτ definition, i.e., {ASIτ, in ms_π_} ~ {ASI, in bits}, is no longer valid.
3. *Other languages*. It has long been noticed that, across languages, syllabic information density (i.e., the average information carried by a syllabic unit, in bits/syllabic-unit) and speech rate (in syllabic-units/s) interact in a negative high correlation. Consequently, a language that carries less information per syllabic unit will “pack” more units per second, e.g., Spanish vs. German (e.g., Pellegrino et al., 2011). How these *source* properties across languages, measured in *nominal* rates (i.e., below capacity) co-exist with our estimate of auditory channel capacity? Following the rational used before, we predict that in a repeat of Experiment I, κ at knee-point of performance (κ^*^) will be such that ϕ^*^ = θ_max_, with a distribution of compressed θ-syllable durations similar across languages, but with language-dependent average ASI (in bits). As such, κ^*^ should be a function of language, with lower values for languages with higher speech rate, e.g., κ^*^_Spanish_ < κ^*^_German_. A corollary to this prediction is that our estimate of auditory channel capacity, expressed in θ-syllables/s, will generalize (to be 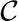
_auditory_ = 9 θ-syllables/s); however, if expressed in bits/s, the auditory channel capacity for German will be greater than that for Spanish.

It is worth emphasizing that our estimate of auditory channel capacity is only valid for young listeners with normal hearing (the age group of our subjects). There is a large variability in how listeners in different age groups perceive time-compressed speech, stemming from either (1) an underlying individual variability in the range of cortical θ, or (2) other deficiencies of neuronal processing at play when listening to time compressed speech. As for the first possibility it may be that, for older adults, the frequency range of neuronal oscillations shifts downward. Therefore, a lower θ_max_ (compared to the young) may result in a reduction in auditory channel capacity. As for the second possibility, some deficiencies were discussed in the previous subsection, “Synthesis by repackaging: acoustics vs. intelligibility.”

### 5.4. Capacity: auditory channel vs. immediate memory

Our way of partitioning the auditory system is shown in Figure 1B. Oscillation-based models exist for both components of the system—the auditory channel and the cortical receiver—with theta oscillations at their core. As is re-iterated throughout the paper, the auditory channel contains oscillation-based functions (e.g., as in Tempo) with theta as master. Immediate memory circuitry, for words, belongs to the cortical receiver (with the lexical-access circuitry the first layer, with pre-lexical units as input and words as output). Recent oscillation-based models of memory circuitry suggest that encoding and retrieval of episodic memory takes place at different phases of theta (e.g., Hasselmo et al., 2002, 2009). Other models (e.g., Lisman and Idiart, 1995; Jensen and Lisman, 1996, 2005), propose neuronal networks with theta cycles at the core, subdivided into seven gamma subcycles. These networks form a short-term memory buffer that can actively maintain about seven memories, in correspondence with the capacity of human's immediate memory (e.g., Miller, 1956). Are the findings of our study—that the auditory channel capacity is determined by cortical theta—reflect channel limitations or the limitations imposed by immediate memory circuitry?

Within the information-theory framework, channel capacity is defined as the maximum information rate, in units of encoder-symbols/s, that satisfies flawless performance measured at the (error-free) decoder. Auditory channel capacity, in particular, is defined as the maximum information rate, in θ-syllables/s, at the knee-point of performance measured at the cortical receiver in word accuracy sense. Thus, the auditory channel output is a sequence of pre-lexical units while the receiver operates on words. We assume an error-free receiver because the behavioral task is a digit-string recognition with a memory load of 4 digits: such memory load is less than the immediate memory span, and the duration of 4 digits is less than the memory decay time (≅2 s, e.g., Cowan, 1984). The assumption of an error-free cortical receiver implies that (1) errors are the result of erroneous pre-lexical units at the channel output (i.e., the errors are induced by the auditory channel), and (2) there are no deficiencies in the immediate memory function (which stores words).

Finally, it is worth noting that, in our view, the theta oscillators in models of the auditory channel are distinct from those in models of the memory. Tempo hypothesizes a special class of oscillators, which allow a gradual change in their frequency while tracking the slowly varying input speech pseudo-rhythm. Such class of theta oscillators is much different from the theta oscillators proposed for memory circuitry, which assume oscillations with fixed, time-independent frequency.

## 6. Summary

Intelligibility of time-compressed 7-digit strings was measured as a function of speech speed and repackaging. Irrespective of speech speed, the maximum information transfer rate through the auditory channel, or auditory channel capacity, is the information in one uncompressed θ-syllable long speech fragment per one θ_max_ cycle, or 9 θ-syllables/s. Interpreted through the prism of oscillation-based models, the alignment of both the packaging rate and the information per packet with properties of cortical theta implies that the auditory channel capacity is determined by theta. We suggest that, in talker-listener communication, the appropriate unit to express speech information transfer rate is θ-syllables/s. Expressed in θ-syllables/s, auditory channel capacity is constant over articulation speed and corpus perplexity (and languages, in particular), equals to 9 θ-syllables/s. Expressing auditory channel capacity in bits/s will result in a source-dependent estimates of capacity.

### Conflict of interest statement

The author declares that the research was conducted in the absence of any commercial or financial relationships that could be construed as a potential conflict of interest.

## Footnotes

^1^These packets are temporally structured so that most of the energy fluctuations occur in the range between 3 and 10 Hz (e.g., Greenberg, 1999; Greenberg and Arai, 2004).

^2^The θ-syllable (Ghitza, 2013), is re-introduced in section “Definitions.”

^3^The Hamming distance between two strings of equal length is the number of positions at which the corresponding symbols are different.

^4^Note that the definition of a mid-vowel location is loose, within a time interval in the order of a few pitch cycles.

^5^Because these simulations are not simply standard error calculations, the credible intervals are not restricted to be symmetrical around the mean, as can be seen under close inspection of the data later on.

^6^Note that we use different superscript symbols to indicate optimum, ^*^ for the compression without repackaging, and ^*o*^ for the compression with repackaging.

^7^Preserving the pitch contour is the main motivation for using the PSOLA methods.
